# Hypomelanosis of Ito presenting with pediatric orthopedic issues: a case report

**DOI:** 10.1186/1752-1947-8-156

**Published:** 2014-05-19

**Authors:** Malene Trägårdh, Christine Rohr Thomsen, Rikke Thorninger, Bjarne Møller-Madsen

**Affiliations:** 1 Department of Orthopaedic Surgery, Aarhus University Hospital, Noerrebrogade 44, DK-8000 Aarhus C, Denmark

**Keywords:** Hypomelanosis of Ito, Orthopedic surgery, Clubfoot, Eczematous rash, Bandage

## Abstract

**Introduction:**

Hypomelanosis of Ito was originally described as a purely cutaneous disease. Extracutaneous manifestations were described later, forming a neurocutaneous syndrome including skeletal, muscular, ocular and central nervous system symptoms.

Hypomelanosis of Ito is characterized by a depigmentation along the lines of Blaschko on the trunk and extremities in certain patterns.

The aim of this article was to report another case and give an overview of the related orthopedic symptoms that have been previously described. It was also our wish to contribute with recommendations for consideration with regard to bandages on eczematous rashes, especially on clubfeet.

**Case presentation:**

A one-and-a-half-month-old boy of Caucasian background born with talipes equinovarus, or clubfoot, on his right foot presented with an eczematous rash after surgical correction and plaster bandaging.

**Conclusions:**

It is the appearance of hypopigmentation, either alone or in combination with a congenital malformation, particularly central nervous system or musculoskeletal anomalies, which should form the basis of a presumptive diagnosis. This should then lead to further investigations and should always include skin biopsies and a test for chromosomal mosaicism.

We report the case of a boy with a clinical picture consisting of a depigmented skin pattern, mental retardation, pes cavus, talipes equinovarus, clinodactyly, eczema, inverted cilia of the eye, strabismus, reduced hearing, ventral hernia, glomerulonephritis, missing testicles, leg length discrepancy with scoliosis, back pain and a syrinx.

It is perhaps impossible to make any conclusions about extracutaneous symptoms. However, some symptoms such as retardation, cramps and seizures, delayed development and hypotonia cannot be ignored.

Because of the possibility of creating an undesirable and long postoperative period with complications, it is very important to have this diagnosis in mind when deciding to do surgery or not if there are signs of dermatological problems before surgery. In this case, it could also be good clinical practice to test the patient’s reaction to plaster or other bandages.

## Introduction

The clinical picture of hypomelanosis of Ito (HI) as we know it today was first described in 1952 by a Japanese doctor named Ito. He described it as ‘incontinentia pigmenti achromians’ and in 1973 Jelinek was able to differentiate this from that of Block-Sulzberger syndrome [1]. Originally described as a purely cutaneous disease, several extracutaneous manifestations were later described and added in the literature, forming a neurocutaneous syndrome including skeletal, muscular, ocular and central nervous system (CNS) symptoms [2,3]. Because of this, the syndrome has been much debated in the literature with regard to whether or not it can actually be called a syndrome or merely should be regarded as a phenotype [4]. The incidence of HI has been reported to be 1:7540 births and the prevalence 1:82 000 individuals in the general population [4]. HI is characterized by a depigmentation of the skin along the lines of Blaschko on the trunk and extremities in whorled and linear streaks and patterns. The lines are formed when the embryo is folded and the cutaneous precursor cells migrate to their destinations. The depigmentation is believed to occur before the 16th day of embryonic development [2]. It is caused by a chromosomal mosaicism, which can occur in almost any autosome or sex chromosome and can also be caused by a nonmosaic balanced X-autosome translocation. Genetic changes, however, are not present in all affected individuals, and it is still a mystery how so many different karyotypes can lead to the same skin phenotype [4,5].

## Case presentation

**Figure 1:**
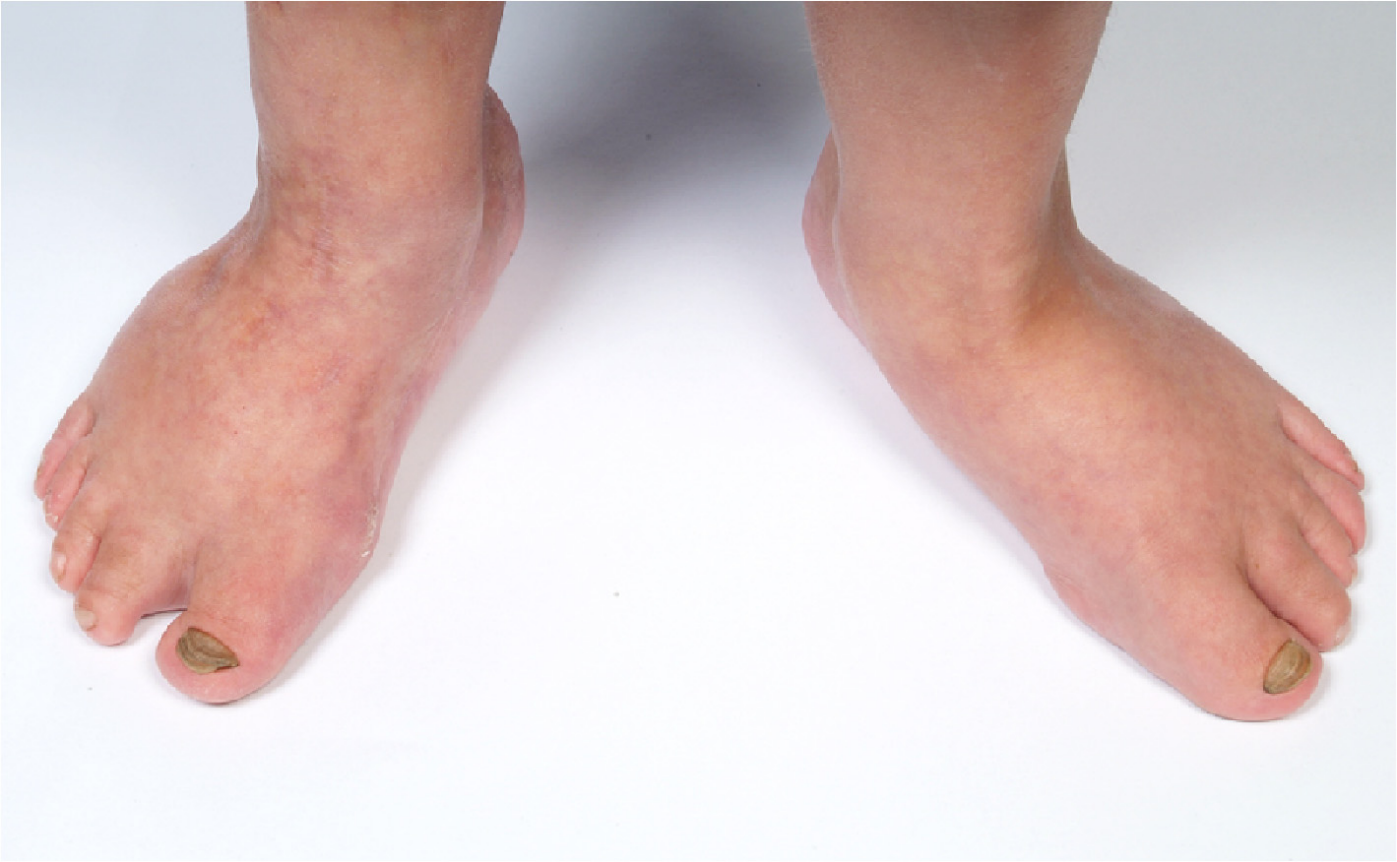
Clubfoot: our patient’s feet showing his postoperative right clubfoot.

**Figure 2:**
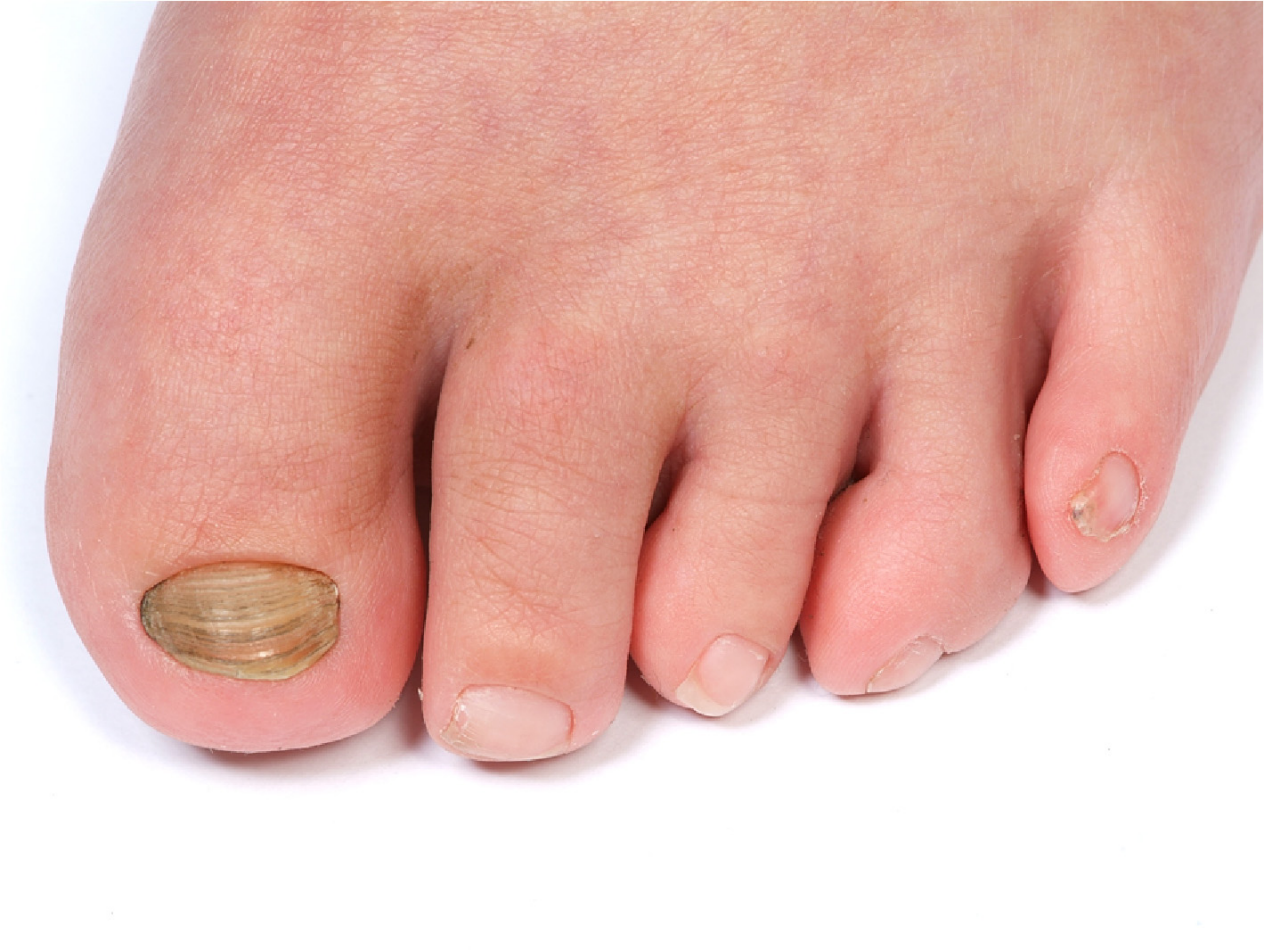
Curly toes: our patient’s left foot showing his curly toes.

A one-and-a-half-month-old boy of Caucasian origins was referred to our hospital for an assessment of his feet. He was born with talipes equinovarus, or clubfoot, on his right foot. Figure 1 shows his right foot postoperatively, after the correction to his clubfoot. On his left foot he had developed pes cavus and he had curly toes on both feet (Figure 2). He had also been treated for a left hip dislocation.

There is no information with regard to the pregnancy itself but the birth was troublesome because he was lying in a lateral position and had to be born with vacuum extraction.

He was born to nonconsanguineous parents and has an older sister who is healthy.

It was found necessary to operate on his right foot and the operation, which went as planned, took place when he was just three months old. It was noted that there was an eczematous rash corresponding to the medial-dorsal skin furrow, which was exactly where the intended incision was to be made. The eczema was described as exuding, with fluid slowly seeping out, but not as a complete epidermis lesion. He was subsequently prescribed peri-operative antibiotics (dicloxacillin) two times per day for the course of one week after the operation, and the eczema was not considered an issue for the success of the operation and healing process.

Ten days after the surgery he was admitted urgently to our hospital because the bandages were foul-smelling and he had failed to thrive for the previous two days. Necrosis was seen around the cicatrix and it was found necessary to revise the wound under general anesthesia. Afterward, he was monitored for a month every other day when he had his bandages changed and the wound healed well. He was fitted with a dynamic splint to support his right foot.

A week after the last control, he was referred from his physiotherapist because of skin problems on his right foot. It was noted that he had a tendency to atopic dermatitis with dry skin and eczema on his cheeks, around the skin lines of his arms and several places on his legs. Furthermore, his skin was described as very delicate, with frequent superficial infections. He now had problems tolerating the splint. There were no general symptoms but it was obvious that his foot was itchy as he was seen constantly rubbing his foot against the mattress.

The eczema developed and spread to both feet between the toes, on the malleoli and on both crura; it was described as an infected asthmatic eczema with dry crusts. He was monitored regularly with regard to the eczema and the splint for the next two years and the skin problem gradually resolved itself. To the best of our knowledge, no allergy test was ever performed testing for allergy to the bandages.

**Figure 3:**
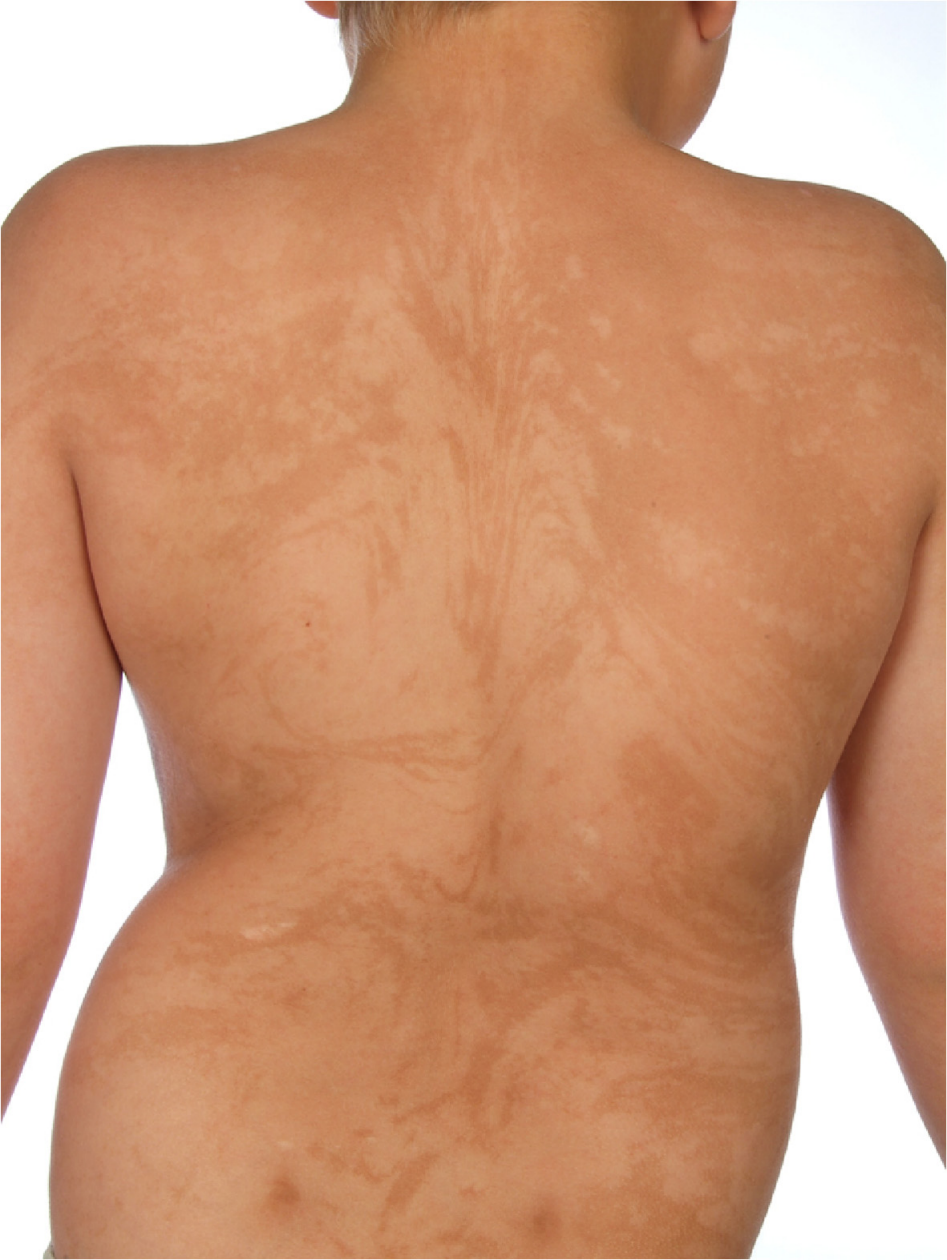
Depigmented skin: the characteristic depigmented skin pattern on the back of our patient.

**Figure 4:**
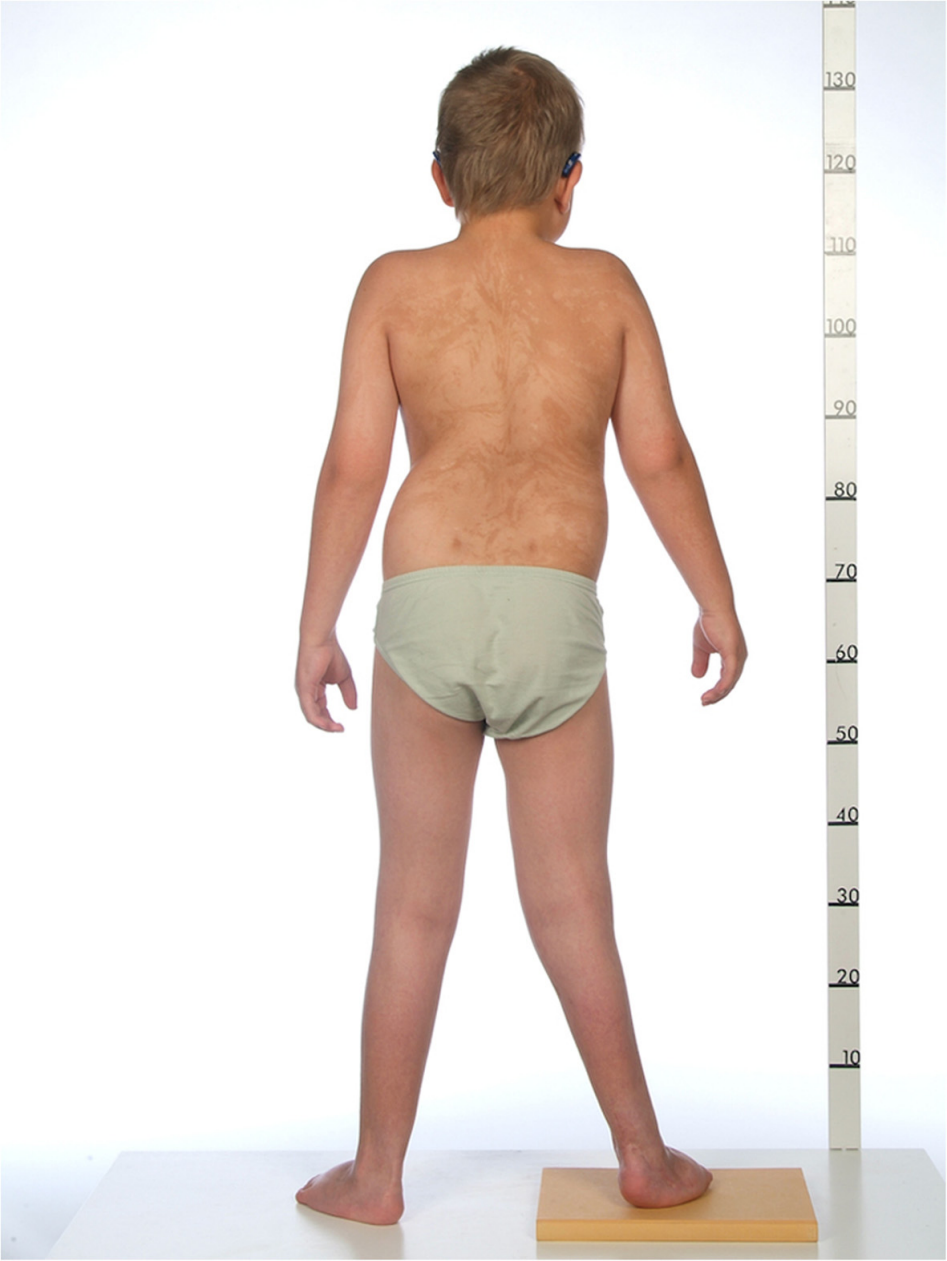
Patient’s back: our patient seen from the back showing the skin pattern, leg length discrepancy of 2 centimetres and scoliosis.

**Figure 5:**
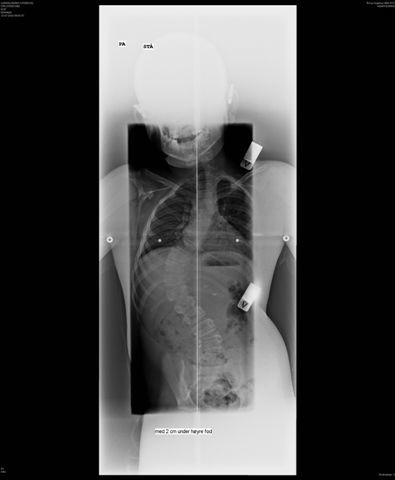
Scoliosis: radiograph showing our patient’s scoliosis.

At the age of two-and-a-half years old, it was noted for the first time that he had a certain type of skin pattern on his back (Figure 3). It had been noted that he had developmental problems and was set back compared to his peers. He was not able to walk independently and his language was remarkably decreased and he was therefore referred to a pediatrician. It was suspected that he had a type of mosaicism but it was not possible to prove the genetic abnormality when skin biopsies were taken. Finally, when he was six years old the pediatrician at a special unit working with rare illnesses made the diagnosis of HI. The diagnosis was based on the criteria listed in Table 1: the clinical picture consisted of the characteristic depigmented skin pattern already noticed at the age of two and five, mental retardation, pes cavus, talipes equinovarus, clinodactyly, eczema, inverted cilia of the eye, strabismus and reduced hearing. He later developed a ventral hernia, which regressed, he got glomerulonephritis with proteinuria at the age of seven and, at the same time, it was discovered that there were missing testicles in both scrota. At the present time, he is 13 years old and has developed leg length discrepancy (Figure 4), back pain and a syrinx corresponding to the conus as well as scoliosis (Figure 5), for which he has recently had successful surgery.

**Table 1:** The diagnostic criteria of hypomelanosis of Ito

**Criterion**	**Features:**
1 (*sine qua non*)	Congenital or early acquired nonhereditary cutaneous hypopigmentation in linear streaks or patches involving more than two body segments.
2 (major)	One or more central nervous system anomalies; one or more musculoskeletal anomalies.
3 (minor)	Two or more congenital malformations other than central nervous system or musculoskeletal. Chromosomal anomalies.
Definitive diagnosis	Criterion 1 plus one or more criterion 2 or two or more criterion 3.
Presumptive diagnosis	Criterion 1 alone or in association with one minor criterion.

The databases of biblioteket.dk, SveMed+, PubMed, Embase, Cochrane Library and Cinahl were searched using the following words, either by themselves or in different combinations: hypomelanosis, pigmentation disorders, hypopigmentation, hypomelanosis of Ito, hypopigmentation [Mesh: NoExp], hypopigmentation [Mesh: narrowed by major subject-skin diseases], dermatology, hip dysplasia, Ito syndrome, orthopedics, orthopedics [Mesh], incontinetia pigmenti.

This search led to the identification of 112 articles, of which the abstracts were reviewed and included based on the following criteria:

– The articles’ main subject had to be HI.

– Written in any of the following languages: English, Danish, Norwegian, Swedish, German and Italian.

After this exclusion of irrelevant articles there were 34 articles left, which we read and from these we chose 10 articles as our main references. The articles were chosen based mainly on these criteria:

– Patients having orthopedic symptoms.

– Patients having dermatological symptoms.

– Articles containing diagnostic criteria and treatment/follow-up suggestions.

The bibliographies of the 10 articles were reviewed in order to discover any other relevant studies.

When constructing Table 2, we used 22 out of the 34 articles and included all case reports in order to obtain the highest number of patients when counting extracutaneous symptoms.

**Table 2:** Extracutaneous symptoms: occurrence of extracutaneous symptoms as reported in 23 articles

**CNS**	**Patients**	**References**
Mental and motor retardation	55	[1,4,7,9-16]
Cramps/seizures	41	[1,4,5,7,10-12,15,17-20]
Delayed motor and mental development	20	[2,11,13-22]
Hypotonia	15	[1,5,11,17-20,22,23]
Microcephaly	12	[1,7,12]
Other	16	[5,7,11,14,15,17-21]
**Musculoskeletal**		
Craniofacial abnormalities	24	[4,5,12,13,17,20,22]
Scoliosis	17	[1,2,6,16,17,23]
Short stature	12	[1,4]
Hemihypertrophy	10	[3,7,10,13,17,19,21]
Clinodactyly	9	[1-3,9,16]
Thorax deformities	9	[1,5,13,21]
Other	39	[1-6,9,11-14,17-23]
**Ocular**		
Hypo-hypertelorism	9	[1,2,9,14-17,22]
Other	35	[1,2,5,7,11,14,15,17,20-23]
**Cardiac**		
Other	6	[1,20,22]

CNS, central nervous system.

Since HI is a very complex diagnosis to make because of the various combinations of nonspecific symptoms described in the literature, it would be advantageous to physicians to have some diagnostic criteria on which they could base the diagnosis.

It is crucial that the patient is born with or develops the characteristic depigmented skin pattern early in life. It should, however, be noted that it can be extremely difficult to see the pattern on fair-skinned individuals. In these cases, a Wood’s lamp can be of help (a diagnostic dermatologic tool with which ultraviolet light can be shone on the patient’s skin, any following fluorescence can then be observed) [6].

In 1992, Ruiz-Maldonado *et al*. [1] published a suggestion for diagnostic criteria. The criteria were composed based on a 20-year prospective protocol performed at a reference center for diagnostic and therapeutic problems of all pediatric specialties. Forty-one cases were included-at the time the largest collection of cases, and therefore based on vast clinical experience. The criteria were again recommended by Failla *et al*. in 1997 [7] and there appears to be no better suggestions until now. The criteria can be seen in Table 1.

For the clinician, it is perhaps not so much the diagnostic criteria as it is the presumptive diagnosis that is more relevant. It is the appearance of the hypopigmentation either alone or in combination with a congenital malformation that should aid diagnosis. This should then lead to an investigation into whether or not the patient suffers from the syndrome. It will always be of utmost relevance to do a skin biopsy from both hypo- and normopigmented skin areas and test for chromosomal mosaicism. It should be remembered though that it is not in all cases possible to verify the diagnosis this way, but that does not mean that the patient does not have HI [8]. It is likely that the problems we are experiencing today with regard to the identification of the mosaicism are merely because of technological limitations and that, in the future, it will be a defining diagnostic criterion.

As the cutaneous and extracutaneous symptoms often reveal themselves early in life, the challenge of diagnosis will often fall on a pediatrician. Other specialities that might be involved in the diagnostic process will often be dermatologists and geneticists because of the cutaneous patterns and combination of syndrome-suspicious extracutaneous symptoms.

Before diagnosing a patient with HI, it is important to have ruled out other hypopigmentation disorders such as the fourth stage of incontinentia pigmenti of Bloch-Sulzberger syndrome, nevus depigmentosus, linear or whorled nevoid hypermelanosis, piebaldism and segmental vitiligo. It must also be kept in mind that certain skin fungi infections can leave a color pattern on the skin, which can be similar to the one of HI [4,7].

In order to clarify if any possible links between HI and any other extracutaneous symptoms exist, the literature was reviewed and case reports including 122 patients were collected, as depicted in Table 2[1-7,9-23].

**Figure 6:**
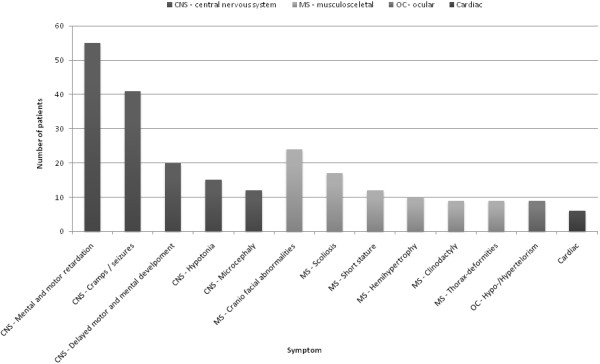
**The distribution of the extracutaneous symptoms listed in Table **2** shown graphically.**

Numerous symptoms are related to the cutaneous manifestations and therefore it is difficult, or perhaps impossible, to reach any conclusions with regard to the linking of HI with any specific extracutaneous symptoms. However, some symptoms cannot be ignored based on the high prevalence, as seen in Figure 6. These symptoms include primarily mental and motor retardation (45 percent), cramps and seizures (34 percent), delayed motor and mental development (16 percent) and hypotonia (12 percent). It may be seen that there is a great similarity between Table 2 and Table 1, although Table 2 contains more specified symptoms.

## Discussion

The diagnosis of HI was introduced in the early seventies and is, therefore, a fairly new diagnosis, with a complexity that seems to widen with every new case found. The diagnosis displays several symptoms, many of which are of orthopedic interest.

This case report shows none of the classic symptoms of HI prior to our patient’s clubfoot operation, apart from a slightly eczematous rash close to where the incision was to be. Despite the lack of evidence of a dermatological illness at the time, our patient developed postoperative skin necrosis and skin problems around the cicatrix for two years after the surgery. It appears that there could have been a link between the intolerance of the bandages and the abnormality of the skin cells.

In one report, a patient developed a streptococcal exanthema only over hypopigmented skin, which reflects the possibility of a correlation between skin affections and the genetic mosaicism in the lines containing abnormal cell clones [9].

In another case, sweat testing of five patients was performed and four were shown to be anhidrotic in the depigmented areas of the skin [10].

## Conclusions

There appears to be a possible connection between the depigmentation caused by genetic mosaicism and other dermatological issues such as in these cases of eczema and anhidrotic skin areas.

Because of the possibility of creating an undesirable and long postoperative period with complications, it is very important in general to have this diagnosis in mind when deciding to do surgery or not. Especially if the patient is very young and still has to show potential depigmentation but presents with dermatological issues such as eczema. The problem might not be the surgery itself but the following bandages.

With regard to the clubfoot in the case of our patient, the alternative to surgery could have been the conservative Ponseti method, where the entire bandage period would have been longer but with more frequent changes, due to the remodeling. This would automatically have created more frequent control of the skin underneath the bandages. It could also be good clinical practice to test the patient’s reaction to plaster or other bandages if they have already shown skin problems before surgery.

## Consent

Written informed consent was obtained from the patient’s next-of-kin for publication of this manuscript and any accompanying images. A copy of the written consent is available for review by the Editor-in-Chief of this journal.

## Abbreviations

CNS: central nervous system; HI: hypomelanosis of Ito

## Competing interests

The authors declare that they have no competing interests.

## Authors’ contributions

MT, CA and RT were major contributors in writing the manuscript. BMM mainly contributed to supervision and editing of the manuscript. All authors read and approved the final manuscript.

## References

[B1] Ruiz-Maldonado R Toussaint S Tamayo L Laterza A del Castillo V Hypomelanosis of Ito: diagnostic criteria and report of 41 cases Pediatr Dermatol 1992 9 1 10 10.1111/j.1525-1470.1992.tb00317.x 1574469

[B2] Horn D Rommeck M Sommer D Körner H Phylloid pigmentary pattern with mosaic trisomy 13 Pediatr Dermatol 1997 14 278 280 10.1111/j.1525-1470.1997.tb00956.x 9263307

[B3] Gartner CM Sabo D Case report of ito-syndrome associated with congenital hemihypertrophy from the orthopaedic point of view Z Orthop Ihre Grenzgeb 2005 143 656 659 10.1055/s-2005-918207 16380898

[B4] Ruggieri M Pavone L Hypomelanosis of Ito: clinical syndrome or just phenotype? J Child Neurol 2000 15 635 644 10.1177/088307380001501001 11063076

[B5] Ruggieri M Roggini M Spalice A Addis M Ianetti P Pigmentary mosaicism, subcortical band heterotopia, and brain cystic lesions Pediatr Neurol 2009 40 383 386 10.1016/j.pediatrneurol.2008.11.006 19380077

[B6] Cellini A Morroni M Simonetti O Oddidani A Hypomelanosis of Ito: a case report with clinical and ultrastructural data J Eur Acad Dermatol Venereol 1998 10 73 76 10.1111/j.1468-3083.1998.tb00933.x 9552763

[B7] Failla P Romano C Schepis C Hypomelanosis of Ito: a syndrome requiring a multisystem approach Australas J Dermatol 1997 38 65 70 10.1111/j.1440-0960.1997.tb01108.x 9159959

[B8] Loomis CA Linear hypopigmentation and hyperpigmentation, including mosaicism Sem Cutaneous Med Surg 1997 16 44 53 10.1016/S1085-5629(97)80035-1 9125765

[B9] Steijlen PM Vietor HE Steensel MV Happle R Sweat testing in hypomelanosis of Ito: divergent results reflecting genetic heterogeneity Eur J Dermatol 2000 10 217 219 10725822

[B10] Shobha N Taly AB Sinha S Arundaya GR Srikanth SG Neurological pictures. hypomelanosis of Ito J Neurol Neurosurg Psychiatry 2006 77 873 10.1136/jnnp.2005.084723 16788014 PMC2117480

[B11] Rutland BM Edgar MA Horenstein MG Hypomelanosis of Ito associated with precocious puberty Pediatr Neurol 2006 34 51 54 10.1016/j.pediatrneurol.2005.06.004 16376280

[B12] Yakinci C Kutlu NO Alp MN Senol M Durmaz Y Budak T Hypomelanosis of Ito with trisomy 13 mosaicism [46, XY, der (13;13) (q10;q10), +13/46, xy] Turk J Pediatr 2002 44 152 155 12026206

[B13] Schmidt H Uhrig S Lederer G Murken J Speicher MR Schuffenhauer S Mosaicism for a dup(12)(q22q13) in a patient with hypomelanosis of Ito and asymmetry J Med Genet 2000 37 804 807 10.1136/jmg.37.10.804 11183189 PMC1757152

[B14] Samuel M Girimaji SR Christopher R Manjunatha: hypomelanosis of Ito Indian J Pediatr 2000 67 473 474 10.1007/BF02859476 10932972

[B15] Pulimood S Rajagopalan B Jacob M George S Korah I Hypomelanosis of Ito with unusual associations Clin Exp Dermatol 1997 22 295 296 10.1111/j.1365-2230.1997.tb01100.x 9604459

[B16] Kukolich MK Althaus BW Freeman MVR Lewandowski RC Hypomelanosis of Ito with triphalangeal thumbs J Med Genet 1980 17 151 152 10.1136/jmg.17.2.151 7381873 PMC1048525

[B17] Schwartz MF Jr Esterly NB Fretzin DF Hypomelanosis of Ito (incontinentia pigmenti achromians): a neurocutaneous syndrome J Pediatr 1977 90 236 240 10.1016/S0022-3476(77)80636-7 830915

[B18] Thapa R Dhar S Malakar R Chakrabartty Hypomelanosis of Ito-whorled hyperpigmentation combination: a mirror image presentation Pediatr Dermatol 2007 24 572 573 10.1111/j.1525-1470.2007.00529.x 17958820

[B19] Sharma S Sankhyan N Kabra M Kumar A Hypomelanosis of Ito with hemimegalencephaly Dermatol Online J 2009 15 12 19951648

[B20] Akefeldt A Gillberg C Hypomelanosis of Ito in three cases with autism and autistic-like conditions Dev Med Child Neurol 1991 33 737 743 1717328 10.1111/j.1469-8749.1991.tb14953.x

[B21] Duran-McKinster C Moises C Rodriguez-Jurado R Tamayo-Sánchez L Orozco-Covarrubias L Ruiz-Maldonado R Streptococcal exanthem in a blaschkolinear pattern: clinical evidence for genetic mosaicism in hypomelanosis of Ito Pediatr Dermatol 2002 19 423 425 10.1046/j.1525-1470.2002.00119.x 12383100

[B22] Boon C Markello T Jackson-Cook C Pandya A Partial trisomy 10 mosaicism with cutaneous manifestations: report of a case and review of the literature Clin Genet 1996 50 417 421 9007335 10.1111/j.1399-0004.1996.tb02399.x

[B23] Happle R Krenz J Pfeiffer R Ito syndrome (incontinentia pigmenti achromians) Hautarzt 1976 27 286 290 950303

